# Navigating family life with Hypoplastic Left Heart Syndrome: A qualitative study

**DOI:** 10.1371/journal.pmen.0000208

**Published:** 2024-12-20

**Authors:** Michael D. Green, Alejandra Prevost-Reilly, Devin M. Parker, Elizabeth Carpenter-Song

**Affiliations:** 1 Department of Population Health Sciences, Duke University School of Medicine, Durham, North Carolina, United States of America; 2 Department of Biological Sciences, Dartmouth College, Hanover, New Hampshire, United States of America; 3 Department of Social Epidemiology, Sorbonne University, Paris, France; 4 Department of Anthropology, Dartmouth College, Hanover, New Hampshire, United States of America; Uganda Martyrs University, UGANDA

## Abstract

Hypoplastic Left Heart Syndrome (HLHS) is a critical congenital heart abnormality that, prior to 1980, offered no treatment options beyond comfort care. Surgical advancements have since transformed the prognosis, yet the lived experience of affected families remains complex and multifaceted. This study aims to elucidate the psychosocial challenges accompanying the biomedical management of HLHS, exploring both family and provider perspectives to identify opportunities for more holistic care. We conducted semi-structured interviews with five families and two healthcare providers involved in HLHS management a New England health system. Interview transcripts were analyzed inductively to identify emergent themes, with a focus on the lived experience of families and the perceived role of providers in influencing this experience. Our study illuminates the extensive psychosocial challenges and emotional distress encountered by families dealing with HLHS, indicating a disparity between the advanced biomedical treatments available and the broader, more integrative care needs of patients. Despite healthcare professionals’ technical proficiency, there exists a pivotal need for empathetic engagement and support that encompasses the full scope of the patient and family experience. Our findings advocate for an integrated care model that incorporates George Engel’s biopsychosocial aspects of health, aligning with the emotional and psychological needs of families. The study underscores the importance of socially conscious care and suggests that enhancing empathetic communication and support in clinical practice can improve both patient outcomes and family well-being in the context of chronic and complex conditions like HLHS.

## Introduction

Until 1980, all children born with Hypoplastic Left Heart Syndrome (HLHS) died within the first week of life. Families had limited options beyond comfort care for their child. The prevalence of HLHS varies based on clinical definition, but it is estimated that it occurs in 1,000–2,000 live births annually in the United States [1–5]. Although HLHS is a rare congenital disability, studies show that it is attributed to approximately 23% of cardiac deaths within the first week of life and approximately 15% within the first month [6]. HLHS is considered the most costly congenital cardiac condition in the United States for individuals and providers, having a direct annual cost estimated at over one billion dollars [7].

Recent surgical innovations have increased survival rates for children with HLHS. The introduction of the Norwood procedure has improved survival from nearly 0% to 42% from 1999–2005 [8]. The current clinical norm in the United States for children born with HLHS involves a three-stage process of interventions: First, the Norwood procedure, performed during the first week of life; second, the Glen shunt, performed around three months after birth; and third, the Fontan procedure, usually performed around the age of three [9]. In non-technical terms, these processes reroute blood flow through the heart, circumventing the individual’s faulty left ventricle. Proper circulation through the Fontan procedure is considered challenging to achieve and has multiple negative long-term consequences due to the new blood circulation pattern that is constructed [10]. Despite these medical advancements, research shows that families often face uncertainty regarding the future requirements of the biomedical system for the ongoing care of their children, or they encounter challenges in meeting their child’s needs [11–22].

Anthropologists use observational ethnographic methods and often incorporate person-centered interviewing [23, 24], to investigate illness experiences. These methods allow them to identify and interpret sociocultural factors that cannot be captured from a quantitative perspective [25]. Moreover, medical anthropologists have argued that the traditional emphasis on identifying and treating biological disorder in medical training and practice may not elucidate meaningful engagement with psychosocial experiences of illness and suffering [26, 27]. As a general trend, biomedical research also lacks detail when analyzing the experience of practitioners who care for patients with chronic illness [28]. To meet this need, we employ anthropological methods to provide insight into the lived experience of five families of children with HLHS, as well as the role of their biomedical providers in influencing their experience.

## Methods

### Ethics statement

Our study design was reviewed by the Institutional Review Board of the health system where the participants were recruited and determined to be “exempt” as defined by Department of Health and Human Services and Food and Drug Administration regulations. Participants provided informed consent verbally, and an optional written consent form was provided prior to the interview. The names of participants, including doctors that advised this project, are replaced with pseudonyms to protect their identity. To make the affiliation of the children and their families easier to follow, we made the first letter of the family name (e.g. “A” in Alston), align with the first letter of the child’s name (e.g. “A” in Andrew” The names of the healthcare centers are anonymized to protect the safety of the practitioners and participants and encourage transparency throughout the interviews.

To comply with the recommendations and maintain exempt status from the Institutional Review Board (IRB), data will not be made publicly available. Requests for deidentified interview transcripts can be made to the corresponding author, or the Dartmouth Hitchcock-Health IRB (IRB ID: STUDY02000542).

### Study design

We conducted seven semi-structured person-centered interviews [23], with five families and two providers (Table 1). Recruitment and interviews took place from July 9^th^, 2020 through December 31^st^, 2020. All recruited participants were the legal guardian and biological parents of a child with HLHS. Families and providers were recruited through an academic medical center in New England by two pediatric cardiologists at that center. These cardiologists were contacted based on their clinical practice and research interests in congenital heart conditions. They were asked about some of their most challenging cases to manage of congenital heart disease. They unanimously identified HLHS as their most challenging condition to manage and agreed to assist us with participant recruitment. Afterwards, they reached out to the parents of all patients with a diagnosis of HLHS within their health system, The listed contact parent with a child with HLHS in the health system were informed of our study, and then study participants contacted us directly or requested that these practitioners provide us their contact information to schedule an interview. After concluding interviews with families, we interviewed the practitioners.

**Table 1:** Pseudonyms of families and physicians interviewed.

**Family Name**	**Parent 1**	**Parent 2**	**Child with HLHS**
*Alston*	Alexa	Zachary	Andrew
*Barton*	Barbara	Bill	Bonnie
*Callan*	Christy	Charles	Catherine
*Davidson*	Donna	Doug	Darby
*Edgar*	Erin	Erick	Emily
**Physician Family Name**	**Physician 1st Name**		
*Knox*	Sheila		
*Grey*	Kathleen		

Each interview lasted approximately one hour and progressed from broad questions to more specific subject matter (Appendices A and B in S1 File) [29]. Our research delineated the lived experience of participants by framing the interview questions around feelings over time and events connected to their child’s condition. Because the investigation of congenital disabilities is extremely reliant on the caregiver during the early years of life when a child is noncommunicative, we relied on parents for insight into the care their child received. To construct our interview guides, (Appendix B in S1 File) we consulted two pediatric cardiologists to identify primary areas of concern for barriers and facilitators to care. These pediatric cardiologists were two of the participants that presented their perspectives as healthcare providers who treat patients with HLHS. All seven interviews were conducted on Zoom video-conferencing software during the summer of 2020 to protect the safety of participants during the on-going COVID-19 pandemic. At the conclusion of the interview, participants were provided $25 Amazon gift cards.

### Theorical orientation

In this study, we examine tensions between biomedical framings of *disease* and the lived experiences of *illness* among families. A biomedical approach defines a patient’s experience by their condition, and limits the role of medical practitioners to treating patients’ physical ailments [30]. Anthropologist Kleinman posits *disease* refers to the biomedical understanding of health. and pathological states. In contrast, *illness* refers to the perception and experiences of states of suffering from the perspective of individuals and families [31]. Kaufman frames the anthropology of illness as the holistic approach that emphasizes sociocultural and behavioral characteristics influencing the care and course of an illness. This distinction reminds us that medicine is a cultural system and provides us with the interpretive framework for this study.

### Analytic approach

For this qualitative study, we used inductive processes to analyze interview data [32]. The interviews were transcribed verbatim and subsequently coded. Qualitative coding is a process used to reduce large volumes of text-based data into manageable units for interpretation [33, 34], by labeling portions of the transcript with words or brief phrases to describe and summarize the meaning of the excerpt. We applied thematic analysis to illuminate emergent themes based on an iterative process of coding, aggregating the data by code, and inductively reviewing the code reports [35]. We used analytic memo-ing [36] to distill key insights into thematic statements, which were concise, precise assertions that encapsulated the essence of emergent themes. Three researchers (M.G., A.P., E.C.) were tasked with independently memo-ing and drafting thematic statements, which were subsequently reviewed and discussed. This process allowed for critical dialogue and iterative feedback, culminating in the refinement of thematic statements to ensure they credibly reflected the intended meaning.

Following this refinement phase to identify key thematic insights, we applied a systematic approach to select the themes of greatest relevance to the study’s objectives. Specifically, we ranked the drafted thematic statements to prioritize themes based on a collective valuation. This approach was supplemented by a notes section in the document where they were aggregated, allowing researchers to justify their rankings, a feature particularly useful in the event of tie scores.

The outcome of this ranking process determined the thematic focus for each section of the results, with researchers assigned to construct initial sections based on the thematic statements that resonated most closely with their analytical insights. This collaborative approach not only fostered a sense of collective ownership over the study’s findings but also ensured that the analysis was grounded in a coherent and rigorously developed thematic framework.

## Results

Thematic Statement #1: *When confronted with severe diagnoses such as HLHS*, *the needs of patients and their families extend far beyond the physical as they grapple with high levels of mental*, *financial*, *and familial strain*.

Families are thrust into making life-and-death healthcare decisions for their newborn child and must simultaneously navigate: quarter-million-dollar medical bills, living at or near the hospital, childcare, and their own health. While successful surgical intervention is crucial to the survival of the patient, families face a suite of other challenges throughout the illness experience that necessitate assistance but often go unaddressed by the biomedical system. Medical practitioners have the potential to meet several of these needs through empathetic and informed biopsychosocial care.

As father Bill Barton describes, a family is given three choices upon their child’s diagnosis: “Heart transplant, three open heart surgeries within the next three years of life or take her home and let her die.” (Table 2, Quote 1) In receiving a diagnosis of HLHS and making this decision, families often undergo a “traumatic experience…not knowing whether they’re going to live or not.” (Table 2, Quote 2) As it felt to the Davidson family, “Life is over…It was literally like the world was falling apart for us.” (Table 2, Quote 3) When a family does make the decision to undergo surgical intervention, it has wide reaching implications for the future lived experience of the whole family. Had the Alston family been told everything they would have to deal with when their child was first diagnosed, they “would have had a breakdown.” (Table 2, Quote 4).

**Table 2:** Supporting quotes for thematic statement #1.

Quote Number	Participant Name	Quote
**Thematic Statement #1: When confronted with severe diagnoses such as HLHS, the needs of patients and their families extend far beyond the physical as they grapple with high levels of mental, financial, and familial strain.**		
1	Bill Barton	Well he gave us three choices, um, heart transplant, um, three open heart surgeries within the next three years real life, or take her home and let her die
2	Christy Callan	It’s a traumatic experience. Um, not knowing whether they’re going to live or not.”
3	Doug Davidson	He’s not going to survive. Life is over. And like, at the same time, like two weeks later, my wife lost her job. Um, and another family thing happened a couple of weeks after that. And it was literally like the world was falling apart for us
4	Alexa Alston	I think if we were sitting at the hospital when he was first born and Dr. [redacted] had sat us down and told us everything that we would be having to deal with I think we would’ve had a breakdown
5	Alexa Alston	I think he was 8 days old when he had his surgery and um we basically lived at the hospital down there…So I was at [redacted]- um the Boston probably maybe a month or not quite a month a little a little probably 3 weeks … looking back it its was pretty devastating at the time but now its kinda part of our world um but he um we lived on the … I slept in the waiting room a lot of nights. I um because I didn’t feel comfortable leaving him
6	Alexa Alston	We ended up getting him in counseling because there was a lot of anger towards his brother he would destroy a lot of his things. Now at the time he was little I disappeared I never been away from him before my pregnancy with and I had and I was gone for over a month. And I think that was hard and he ended up living with my parents for a while because they were helping out it was good my parents were good to him but it wasn’t, the same I think he had a little anger. Its hard for a little one to understand all of that
7	Alexa Alston	It’s exhausting, um, applied for social security, Medicaid, all that stuff was a lot of work. And then they were charging the wrong [insurance company] because we were down in [a different state] and that get confused. And so we were getting bills for $300,000, which I knew we didn’t have, but again, you can’t just say, Oh, they don’t mind to pay
8	Alexa Alston	However anxiety wise as a parent I am the only one who carries insurance in my house and so for me knowing that if anything god forbid happens to me um there’s huge anxiety around that my son won’t be covered and its very expensive um so I mean that’s always in the back of my head.
9	Doug Davidson	I think one of the things I wanted so badly, and I still want to this day is for people just to say, like, it’s not fair that you guys are going through what you’re going through and I’m really sorry. Like, I’m really sorry that you have to face this. I’m really sorry that you don’t know if [redacted] is going to live to be 20. I don’t, I’m sorry. If you don’t know he’s going to live to be 30. That’s really hard. Do you want to talk to me about it? Can I listen to you? Can I hear where you’re coming from? Like just empathy
10	Dr. Kathleen Grey	Do you tell them all the complications that could possibly happen? No. Um, but which ones do you pick and choose? And, and sometimes it’s hard when you’ve only been just introduced to the family to know how much really, to, to tell them how much do you worry in […] So, um, yeah, the complications and the unknown of what’s going to happen is definitely the hardest because I do have kids who do have died from the condition. So, um, that unknown is really hard.

As their child undergoes surgery, family members are bound to the hospital for weeks at a time. Often receiving medical care several hours from home, many parents spend nights sleeping in the waiting room or on the floor, “basically living at the hospital” (Table 2, Quote 5). Alexa Alston detailed the impact her time away from home had on her family, describing how hard it was for her older child to have his mother gone for weeks at the hospital. Alexa ended up seeking counseling for her older son because of the anger he had towards his younger brother with HLHS. This exemplifies the need these families have for care outside the surgeries their child with HLHS received, specifically care with a psychosocial lens that can address their emotional needs.

In addition to emotional and familial strain, financial stress was a burden for several families. The Barton family described the laborious task of applying for social security, Medicaid, and sorting out their insurance. They described the exhaustion, confusion, and anxiety that came with working through all these funding sources and still receiving bills for 300,000 dollars because of an insurance mix up. Another parent stated being the only insurance carrier in their household as a major source of anxiety. (Table 2, Quote 8) They were constantly worried that if something happened to them, their son’s medical costs would not be covered. Navigating the financial intricacies and barriers of the United States healthcare system while simultaneously grappling with the enormous unknown of their child’s future was overwhelming for families and further compounded the stressors shaping their lived experience.

The experiences of these families demonstrate the multifaceted nature of the illness experience and reveal opportunities for the biomedical system to improve the lives of families through a more holistic biopsychosocial approach [37]. While a highly skilled surgeon and advanced biomedical technology are required to medically treat a patient, these components alone can fall short at addressing the comprehensive needs of patients and their families. Social services have the potential to play a very large role in filling these gaps, but clinicians can also play a role in caring for families’ psychosocial needs through providing care which is aware and responsive to the broader social factors effecting health. As Doug Davidson describes, “One of the things I wanted so badly, and I still want to this day, is just for people to say it’s not fair that you guys are going through what you’re going through and I’m really sorry…Do you want to talk to me about it? Can I listen to you? Can I hear where you’re coming from? Like just empathy.” (Table 2, Quote 9) The role of empathy in patient-physician interactions had a powerful impact from the perspective of families of children with HLHS. While it will not technically be the factor that saves the child’s life, it has profound effects on how families cope with the uncertainty, anxiety, and strain they are subject to throughout the whole illness experience, and enables them to feel seen, supported, and understood.

Thematic Statement #2: *Families relied upon highly skilled healthcare professionals to competently intervene to address their child’s biological needs and deeply valued healthcare providers who combined technical expertise with empathy*.

As families navigated their children’s serious health conditions, they interacted with a range of highly skilled healthcare providers. Parents in the study valued, and relied upon, the technical expertise of healthcare professionals to perform the complex surgeries required to sustain life. These healthcare providers were lauded as “amazing” and “incredible” in terms of their knowledge and skill. At the same time, parents spoke extensively about the qualities beyond biomedical expertise that mattered to them in the care of their children. In the context of fear and uncertainty about their children’s survival and long-term wellbeing, parents expressed the importance of feeling supported, known, and cared for as a family by healthcare providers. (Table 3, Quote 1) Parents also appreciated when providers viewed their children holistically as a person, not just a disease to be treated. (Table 3, Quote 2) Others noted positive experiences with healthcare providers who took time with families to “truly listen” to their concerns and questions (Table 3, Quote 3) and who communicated with compassion and clarity (Table 3, Quote 4). Healthcare providers demonstrated empathy through concrete manifestations of their concern. In one particularly poignant example, a parent recounted observing the physician comforting her newborn daughter in the middle of the night following surgery. (Table 3, Quote 5) Parents contrasted these positive experiences with instances of feeling “scared” by healthcare providers who, for example, “took away all our hope” (Table 3, Quote 6) and those who were “arrogant” or minimized their concerns (Table 3, Quote 7).

**Table 3:** Supporting quotes for thematic statement #2.

Quote Number	Participant Name	Quote
**Thematic Statement #2: Families relied upon highly skilled healthcare professionals to competently intervene to address their child’s biological needs and deeply valued healthcare providers who combined technical expertise with empathy.**		
1	Alexa Alston	she actually knows my history and…she’ll come in and she’ll put her finger on [my son], checks him out and then she kinda checks me out because she knows I have anxiety …she also checks the health of the whole group around him and that was the same with [other physician]. I always felt that he understood us as a family and that he kind of had this finger on everything. So and those are the ones that are good.
2	Alexa Alston	when he was born and we were dealing with [physician], one of the things he said to us Was, ‘He’s a child first, he’s a kid with a heart condition second. Treat him like a child
3	Doug Davidson	we know that she’s like a super busy doctor… but she will sit in the exam room with us for a half hour, 45 minutes and answer any question we have. …no matter how many questions we have, she’ll still listen and she’ll hear everything we have to say
4	Bill Barton	he told us the worst news and he told it in a way with compassion, respect, and he explained it very simply so we could understand it.”
5	Dr. Sheila Knox	I think HLHS has been, um, something we’ve been thinking about in our community for a long time and how to do a better job of it. Um, obviously, uh, over the last 20 to 30 years of taking care of these patients, even 40 years almost, I mean, there has been a lot of improvement in terms of how to care for them medically and surgically, and to really improve our survivals, uh, for the, for the patients. […]

Thematic Statement #3: *Relationships forged beyond clinical boundaries*, *between healthcare providers and families*, *embody socially conscious care*. *This approach not only enriches the healthcare experience for families dealing with Hypoplastic Left Heart Syndrome but also fosters a sense of community and ongoing support*.

In managing HLHS, the significance of clinical relationships that extend beyond conventional medical interactions becomes evident. Our findings indicate that these relationships, characterized by empathy and personal engagement, play a crucial role in the overall healthcare experience of families dealing with HLHS.

One striking example is detailed by Doug Davidson, who describes a meaningful interaction with a sonogram technician (Table 4, Quote 1). The technician’s thoughtful gestures and constant comradery resonated deeply with the Davidson’s, enabling them to feel truly cared for in a biomedical setting. These actions reflect a deep commitment to patient well-being and signify a personal investment that augments the traditional clinical relationship. An experience with a doctor not interviewed in this study, shared by Bill Barton, further highlights the importance of empathy in clinical practice. This doctor communicated the diagnosis of HLHS with compassion, respect, and clarity, focusing not only on the transmission of medical information but also on the manner of its delivery, significantly impacting the family’s experience at a vulnerable time (Table 4, Quote 2). Doug Davidson had similar reflections on Dr. Grey’s practice, which contrasted sharply to his experience of other medical professionals. Dr. Grey’s approachability and empathetic listening skills, as opposed to a more detached or authoritative style demonstrated by others, influenced the family’s sense of trust and comfort (Table 4, Quote 3).

**Table 4:** Supporting quotes for thematic statement #3.

Quote Number	Participant Name	Quote
**Thematic Statement #3: Relationships forged beyond clinical boundaries, between healthcare providers and families, embody socially conscious care. This approach not only enriches the healthcare experience for families dealing with Hypoplastic Left Heart Syndrome but also fosters a sense of community and ongoing support.”**		
1	Doug Davidson	But the echo tech is a sonogram tech or whatever. Um, at, at [redacted] literally like her name’s [redacted] and she’s incredible. She, my wife has developed a friendship with her and when she went on vacation last year, bought like a specific present for [redacted]. And like the last two times she’s been on vacation. Like she buys something for [redacted] and next time we come in, she has a present for him
2	Bill Barton	We met with Dr. [redacted] and he probably is, uh, one of the most influential, uh, positive, uh, great man that he was the right guy at the right time for us. Um, he sat us down and there was an intern as well. I believe he had and told us the worst news and he told it in a way with compassion, respect, and he explained it very simply so we could understand it
3	Doug Davidson	But no matter how many questions we have, she’ll still listen and she’ll hear everything we have to say. So that’s, um, I think that’s, that’s like the biggest thing, like, and just her. Oh, I dunno just kinda like her demeanor. Like, she’s very like, soft-spoken like, this is, I don’t know if you’re training to be a doctor, so please don’t be offended by this. But like, there’s a lot of, uh, just kind of, uh, full of themselves doctors who thinks that like everyone should just bow down to what it is they say, because they’re a doctor and, and they are in genius. People who keep my son alive and I do not have anything against them in that way, but Dr. [Grey] is much more just like a approachable individual who you can tell truly listens and, um, understands, I think in some ways where we’re coming from
4	Alexa Alston	Um and I should’ve believed myself but I didn’t and we took him to the then pediatrician we had who after being home only a week yelled at us about being overprotective parents and that he was just a colicky baby. Um and that was Sunday night and so Monday morning I was going down to [redacted] anyway for follow-up with Dr. [redacted] and when we got there for an eight o’clock appointment and by then my son had become so dehydrated because of an infection they felt that he had around his heart he blew a clot
5	Dr. Sheila Knox	I think as physicians, we always give a more rosy and hopeful picture to our patients because that’s what we, uh, that’s what we want them to perceive. And we, and we do feel that too, like we, we do present the fact that things have come a long way and that we’ve, we’ve improved a lot, um, in terms of how we presented to them. I think we tell them the numbers. We tell them the facts about what the true survivals are, what the rates of complications are. Uh, we tell them, though, that every kid is individual. And so that, you know, even though we say 85% survival, that doesn’t help when it’s just your kid.

However, not all interactions between healthcare providers and families are positive. Alexa Alston’s experience illustrates the harmful consequences of unsympathetic and dismissive attitudes from healthcare providers. After returning home with their child, Alexa sought help from their pediatrician, concerned about their child’s condition. Instead of receiving support, they were criticized for being “overprotective parents.” This negative encounter led to a delay in seeking necessary care, resulting in their child’s condition worsening due to dehydration from an infection. The lack of empathy and understanding from the pediatrician had significant repercussions, emphasizing the need for healthcare providers to avoid prejudiced judgments and to listen attentively to parents’ concerns (Table 4, Quote 4).

These accounts collectively underscore an often-undertrained and deprioritized aspect of care-delivery dimension in clinical care–the importance of human connection and socially conscious care through communication. In chronic and complex conditions like HLHS, where the journey involves numerous medical challenges and uncertainties, the support from healthcare providers who engage on a personal level can be a significant factor in enhancing the overall healthcare experience for families. This support contributes to their emotional and psychological well-being, supplementing the medical management of the condition.

## Discussion

Our study sheds light on the extensive psychosocial challenges families faces in the context of HLHS, alongside the clinical hurdles of this severe diagnosis. The traumatic experiences associated with critical decision-making processes and their subsequent impact on family dynamics and mental health were vividly detailed by participants. These narratives reveal a significant gap in the current healthcare model, highlighting the on-going need for a comprehensive biopsychosocial approach that encompasses the entire spectrum of family needs throughout the HLHS treatment journey. While the technical expertise of healthcare professionals is essential, our findings emphasize the equally vital role of empathetic engagement and support. This support should extend beyond the diagnosis to acknowledge the full scope of patient and family experiences. Effective communication, psychosocial support, and genuine empathy from healthcare providers are integral to the therapeutic process, profoundly influencing family coping mechanisms and overall well-being.

Our findings advocate for an integrated care model that elevates these interpersonal aspects to a central role in HLHS management, underscoring the significant impact of healthcare interactions characterized by personal investment, empathy, and respect. Such interactions can greatly enhance the treatment experience or, if lacking, severely undermine it. The marked contrast we observed between positive clinical relationships and those marred by dismissal and misunderstanding highlights the pivotal role of healthcare providers in shaping patient and family outcomes. This dichotomy signals a pressing need for systemic reforms aimed at promoting empathetic, patient-centered care within the biomedical context. It affirms the essential role of healthcare systems in providing comprehensive support to families as they navigate the complexities of HLHS, suggesting that a shift towards a more holistic, empathetic approach in healthcare could improve the experiences and outcomes for these families.

All the families participating in our study opted for intensive surgical interventions to address their child’s HLHS, a decision demonstrating their reliance on and trust in biomedical solutions and practitioners. Medical training traditionally emphasizes the physiological aspects of human health, focusing on observable cause and effect for patient treatment and care, viewing these elements as concrete and objective [38, 39]. Yet, the lived experiences of patients and their families extend well beyond the confines of physiological conditions, encompassing a wide array of psychosocial factors that significantly influence their journey through illness and care.

The process by which practitioners earn the trust of their patients and deliver care that is both informed and empathetic is crucial to the overall experience within the biomedical system. This emphasizes a pivotal shift needed in biomedical practice: moving beyond a purely physiological focus to embrace a more holistic approach that considers the emotional, social, and psychological dimensions of patient and family experiences. Such an approach not only aligns with the principles of patient-centered care but also recognizes the intricate interplay between the biomedical and the biopsychosocial aspects of health, advocating for a care model that fosters trust through empathy and consideration of social factors alongside clinical factors.

Biomedicine traditionally centers on a singular cause, leading physicians to deliver diagnoses that are clinically one-sided and objective, often overlooking the nuanced dialogue with patients and families [38]. This approach, however, falls short in the context of HLHS, where physicians must navigate a more intricate landscape. Conveying a diagnosis of HLHS to a family involves presenting them with profoundly difficult choices: either to embark on aggressive, high-risk medical interventions or to face the heart-wrenching decision of not pursuing treatment. Such moments require physicians to extend beyond clinical diagnostic roles to become compassionate communicators and advisors.

While biomedical practitioners possess the skills to perform life-saving procedures for children with HLHS, comprehensively addressing the condition’s wider life implications for patients and their families is not typically covered in conventional biomedical education. The call for a more holistic approach in biomedicine is clear, one that incorporates a greater focus on the psychosocial elements that significantly influence the illness experience [37]. Healthcare providers, therefore, must balance the delivery of clinical facts with providing emotional support, guiding families through high-stakes decisions shrouded in uncertainty. This dual responsibility underscores the need for a healthcare paradigm that fully embraces the complex human dimensions of medical care, ensuring that the path chosen by families is navigated with empathy, understanding, and comprehensive support.

Healthcare providers must often transcend the confines of their biomedical training to truly connect with the lived realities of patients and families. In the case of HLHS, the capacity for empathetic engagement by physicians and healthcare providers becomes vital, linking medical competence with compassionate care [40]. This study’s providers exemplify the biopsychosocial model, which acknowledges that patient and family needs extend beyond the physical; their psychosocial well-being is pivotal to the illness experience [37]. While the diagnosis of HLHS is frequently made prenatally, thanks to advanced medical technologies that epitomize the capabilities of biomedicine [38], the journey for patients and their families is profoundly complex and multifaceted. The technical achievements of biomedicine, while remarkable, represent only a portion of the care required by these families. The treatment trajectory for HLHS, though technically specific, must be navigated with an equal measure of dignity and autonomy afforded to patients and their families. It is imperative to recognize that the implications of physical treatment are deeply intertwined with emotional and social considerations, all of which collectively shape the family’s overall experience within the biomedical system. Thus, the pursuit of medical excellence must be balanced with the delivery of care that honors the holistic nature of the patient and family journey.

Throughout our study, families consistently reported positive interactions with their practitioners, underscoring the pivotal role of biomedicine in managing high-stakes illness experiences. Notably, despite the potential for such intensive medical regimens to feel impersonal or overwhelming, the families did not perceive their interactions with the biomedical system as dehumanizing. This can be largely attributed to the empathetic and biopsychosocial approach adopted by their physicians, which enabled the families to benefit from the medical system’s offerings while preserving their sense of agency and personal dignity.

While practitioners adeptly convey the physiological details of HLHS, the broader impact of the condition on the day-to-day lives of families often remains a more daunting task. Technological prowess in biomedicine is celebrated for its healing potential [41], yet it is not a universal remedy for the diverse factors that shape the illness experience. The empathy demonstrated by practitioners, reflected in their personal commitment to patient care, is fundamental to building and maintaining trust within the biomedical system. However, traditional biomedical training does not always equip physicians with the tools to navigate the uncertainties that families face [42]. More comprehensive training focused on communicating complex and sensitive health information could better prepare physicians for these challenging conversations. In addition, training in social determinants of health and trauma-informed approaches is needed to equip physicians with key knowledge and skills to navigate the complex psychosocial challenges that families face [43].

The personal involvement of physicians with families—a trait highly valued by our study participants—highlights the capacity of providers to deliver care that is both technically proficient and deeply compassionate. Physicians like Dr. Grey and Dr. Knox exemplify this quality, yet it is unrealistic to expect all practitioners to naturally possess such a blend of expertise and empathy. Conditions like HLHS demand clear communication and genuine empathy, particularly in moments of crisis. Current biomedical training, with its focus on treating physiological ailments, may not sufficiently prepare practitioners for the complex, emotionally charged aspects of care. A paradigm shift in healthcare, toward a model that equally addresses the psychosocial aspects of illness alongside the physical disease, could profoundly enhance care for patients and their families, ensuring a more holistic and compassionate healthcare experience.

### Limitations

This study employed remote research methods to engage with participants, a strategy that facilitated the inclusion of individuals across diverse geographical locations without the need for travel. However, this approach inherently excluded those without the necessary technological means to participate in remote interviews. There is a small sample of individuals whom were interviewed, which is expected given the rare nature of this condition. Furthermore, the design of our study meant that the insights gathered were limited to the experiences of families who chose to pursue surgery for their children with HLHS. Consequently, the perspectives of families whose children did not survive or who chose not to pursue the initial three surgeries intended to extend life remain unrepresented. Future research should aim to capture these missing narratives to provide a more comprehensive understanding of the family experience in the context of HLHS. Additionally they should attempt to document the experiences of as many individuals as possible to create a representative sample of those whom experience this condition.

### Conclusion

This exploration illuminated the profound engagement families have with the biomedical system, marked by acceptance and extensive interaction with its mechanisms. In navigating this complex landscape, which includes multiple high-stakes surgeries, interactions with a variety of medical specialists, and the intricacies of healthcare access, families face the added challenge of negotiating care within a healthcare system characterized by a diverse array of insurance frameworks and provider networks, both private and public. To better support these families, it is imperative that medical education adopts a more holistic approach, one that equips practitioners with the skills to provide care that comprehensively addresses the multifaceted nature of illness experiences beyond the biomedical model.

## Supporting information

S1 File Appendix A, full interview guide for parents of children with HLHS. Appendix B, full interview guide for pediatric cardiologists.
(PDF)
