# Supplementary material for: Navigating family life with Hypoplastic Left Heart Syndrome: A qualitative study
Source: PLOS Ment Health. 2024 Dec 20;1(7):e0000208. doi: 10.1371/journal.pmen.0000208 (PMC12199268; doi:10.1371/journal.pmen.0000208)
Supplement: S1 File — Appendix A, full interview guide for parents of children with HLHS. Appendix B, full interview guide for pediatric cardiologists. (PDF) [file pmen.0000208.s001.pdf]

Supplementary Materials

Appendix A. Family Interview Guide

-I wish to begin this interview learning a little bit about the background of your family. Tell me a little bit about yourself

-Where are you from?

-What is your household composition?

-What type of insurance do you hold?

-Can you describe the time when you were informed that your child has HLHS?

  -How long has your child been seeking care?

  -Did you consider this a severe condition?

  -What type of information were you provided initially?

-Has your families financial well-being or employment status been affected from your child's condition?

-How would you describe HLHS to someone unfamiliar with the condition?

  -Has your perception of HLHS changed over time?

-Can you describe what caring for your child's condition looks like for your family?

-What additional services have you needed to care for your child in addition to with the cardiologist (some examples are home nursing, therapies, educational, social work, financial support)

**Follow-up: Are there any services that you wish were available to help better care for your child?

-How have you and other individuals in your family been affected by this condition personally?

  -*(if applicable)*

    -How has your spouse been affected?

    -Other children?

    -social life?

-How has your families' life been affected over time that you consider a result of HLHS?

-What has been your experience seeking care at Dartmouth-Hitchcock Health?

-Are there any experiences you consider unique to Dartmouth-Hitchcock Health that you think are unique to your setting?

-What factors influence the ability for your child to receive care?

-What would you consider a barrier to accessing medical care?

  -How does cost of care influence your experience seeking care?

-Have you experienced barriers to accessing medical care at Dartmouth-Hitchcock?

-Have you received any support coordinating your child and families needs?

  -If not what type of support would you wish to receive?

-Can you compare your initial discovery of your child's condition to your currently outlook?

  -What changed the most?

  -What do you wish you were informed of retrospectively?

  -What will be more helpful for the future?

Is there anything else you would like to tell us?

Appendix B. Provider Interview Guide

How do you perceive HLHS?

- Is it an emerging threat, or are complications manageable?
- Is it something that you are particularly concerned about?

How do/would you translate that perception to families with children with the condition?

- what parts are the hardest for you to explain?
- what parts are the easiest for you to explain?

What do you perceive as barriers to care for families?

- do you believe that any of these barriers are more significant than others?
- what contribute to how these barriers form?

What are some factors that influence your ability to provide care for families?

What is necessary for the future, for families to have better experience?

Is there anything else you would like me to know?
